# Health Care Providers’ Readiness to Adopt an Interactive 3D Web App in Consultations About Female Genital Mutilation/Cutting: Qualitative Evaluation of a Prototype

**DOI:** 10.2196/44696

**Published:** 2023-09-28

**Authors:** Olivia May Holuszko, Jasmine Abdulcadir, Daisy Abbott, Jennifer Clancy

**Affiliations:** 1 School of Simulation & Visualisation Glasgow School of Art Glasgow United Kingdom; 2 College of Medical, Veterinary and Life Sciences University of Glasgow Glasgow United Kingdom; 3 Department of Women, Child and Adolescent Faculty of Medicine University of Geneva Geneva Switzerland; 4 School of Medicine, Dentistry and Nursing College of Medical, Veterinary and Life Sciences University of Glasgow Glasgow United Kingdom

**Keywords:** FGM/C, 3D, interactive, patients, consultation, web app, health care provider, female genital mutilation or cutting, vulva, anatomy

## Abstract

**Background:**

Comprehensive and appropriate health care provision to women and girls with female genital mutilation or cutting (FGM/C) is lacking. Use of visuals in health care provider (HCP) consultations facilitates the communication of health information and its comprehension by patients. A web app featuring a 3D visualization of the genitourinary anatomy was developed to support HCPs in conferring clinical information about FGM/C to patients.

**Objective:**

The aim of this study was to explore HCP perspectives on the use of visuals in discussion about FGM/C with their patients as well as to obtain their feedback on whether an interactive 3D web app showing the genitourinary anatomy would be helpful in patient consultations about FGM/C, identifying key features that are relevant to their clinical practice.

**Methods:**

We evaluated the web app through a semistructured interview protocol with seven HCPs from various disciplines experienced in care for women and girls with FGM/C in migration-destination settings. Interviews were audio- and video-recorded for transcription, and were then analyzed thematically for contextualized data regarding HCPs’ willingness to use a 3D web app visualizing anatomy in FGM/C consultations with patients.

**Results:**

All but one of the seven participants expressed keen interest in using this web app and its 3D visuals of anatomy in FGM/C consultations with patients. Participants shared the common contexts for the use of visuals in health care for FGM/C and the concepts they are used to support, such as to help describe a patient’s genitals after FGM/C and reinforce an understanding of clitoral anatomy, to illustrate the process of defibulation, or to explain the physiological effects of FGM/C. Participants also highlighted the benefit of using visuals that patients can relate to, expressing approval for the ability to customize the vulva by FGM/C subtype, skin tone, and complexity of the visual shown in the web app. Despite critiques that the visualization may serve to perpetuate idealistic standards for how a vulva should look, participants largely agreed on the web app’s perceived usefulness to clinical practice and beyond.

**Conclusions:**

Evaluation of the web app developed in this study identified that digital tools with 3D models of the genitourinary anatomy that are accessible, informative, and customizable to any specific patient are likely to aid HCPs in communicating clinical information about FGM/C in consultations. Universal access to the web app may be particularly useful for HCPs with less experience in FGM/C. The app also prompts options for applications such as for personal use, in medical education, in patient medical records, or in legal settings. Further qualitative research with patients is required to confirm that adoption of the web app by HCPs in a consultation setting will indeed benefit patient care for women and girls with FGM/C.

## Introduction

Female genital mutilation or cutting (FGM/C) is a violation of human rights that affects hundreds of millions of women and girls worldwide; yet, health care provision to those living with FGM/C often remains inadequate [1-8]. Practiced in over 30 countries in Africa, Asia, and the Middle East, as well as among certain ethnic groups living in high-income countries, there is an increasing number of patients with FGM/C living in the diaspora as a result of international migration patterns [9]. Nevertheless, many health care providers (HCPs) around the globe lack the appropriate training and understanding of FGM/C required for effective care and communication, in addition to the language barriers often existing between patients and providers in migration-destination settings [4,10-12]. Clinical information is not always effectively transmitted, leaving patients with FGM/C excluded from decision-making regarding their own health care and treatment [4,10]. Researchers continue to debate the most appropriate ways of improving communication and delivering important health information about FGM/C to patients and their families, with the goal of supporting the women and girls already living with FGM/C while striving toward prevention of the practice [4,13-15].

FGM/C is commonly distinguished according to the typology determined by the World Health Organization (WHO), which describes the extent of cutting to the external genitalia into four main types that can be further subclassified according to the genital structures involved (Table 1) [16]. While categorization by HCPs is an important component of medically recording and reporting FGM/C to relevant authorities, difficulties arise due to the heterogeneity of FGM/C and its practice. Likewise, the health impacts of FGM/C differ from person to person, and health care provision requires a personalized, multidisciplinary approach that caters to each person’s individual experience in a nonjudgmental, supportive, and culturally sensitive manner [16-18].

**Table 1:** World Health Organization descriptions of female genital mutilation or cutting (FGM/C) classification by type, updated from previous guidelines to specify cutting of the clitoral glans as opposed to total removal of the clitoris [9].

FGM/C type		Description
**Type I: Partial or total removal of the clitoral glans (clitoridectomy) and/or the prepuce**		
	Type Ia	Removal of the prepuce/clitoral hood (circumcision)
	Type Ib	Removal of the clitoral glans with the prepuce (clitoridectomy)
**Type II: Partial or total removal of the clitoral glans and the labia minora, with or without excision of the labia majora (excision)**		
	Type IIa	Removal of the labia minora only
	Type IIb	Partial or total removal of the clitoral glans and the labia minora (*prepuce may be affected*)
	Type IIc	Partial or total removal of the clitoral glans, the labia minora, and the labia majora (*prepuce may be affected*)
**Type III: Narrowing of the vaginal opening with the creation of a covering seal by cutting and appositioning the labia minora or labia majora with or without excision of the clitoral prepuce and glans (infibulation)**		
	Type IIIa	Appositioning of the labia minora
	Type IIIb	Appositioning of the labia majora
Type IV: Other		All other harmful procedures to the female genitalia for nonmedical purposes, such as pricking, piercing, incising, scraping, and cauterization

FGM/C is not associated with any known health benefits, but the practice increases the risk of suffering from short- and long-term consequences that may affect physical, mental, and social well-being [16,19]. The procedure itself can also result in life-threatening complications, which may depend on the context, type of FGM/C, and method by which it is performed [19]. Despite the scarcity and low quality of evidence to support guidelines for the clinical care of patients with FGM/C, recommendations and best-practice statements exist around managing patients’ genitourinary, reproductive, sexual, and psychological health [16,20]. These include defibulation (also known as “deinfibulation”), a surgical procedure that opens the vestibule with the vaginal and urinary openings in patients with FGM/C type III to improve overall well-being, allow sexual intercourse, and facilitate childbirth; psychological support, including cognitive behavioral therapy following FGM/C or its surgical management for patients experiencing anxiety, depression, or post-traumatic stress disorder; sexual counselling to prevent or treat sexual dysfunction; and access to health education around FGM/C and women’s health in general, including clear communication of available treatment options [16,20]. Some women may request “clitoral reconstruction surgery,” which should be offered as part of multidisciplinary care alongside sexual counselling [9]. Clitoral reconstruction consists of removing the scar above and around the clitoris to re-expose healthy clitoral tissue in a more visible and accessible position. The surgery and associated psychosexual support can improve women’s sexual health, body image, and eventual clitoral pain, as well as answer to the desire of feeling symbolically “repaired” [9,21].

Shared decision-making in the clinical management of FGM/C, per the guidelines above, requires that patients are provided with clear, evidence-based resources and the opportunity to make informed decisions about their treatment and their body. Furthermore, the first step in conveying health information to and providing clinical care for patients living with FGM/C is ensuring that HCPs themselves have the relevant knowledge, information, tools, and ability to communicate effectively with their patients [22]. While HCPs’ roles in delivering multidisciplinary care to women and girls with FGM/C may vary in scope, patient consultations are a key component of clinical practice, with discussion and exchange sometimes facilitated by the presence of a certified interpreter or cultural mediator [9].

Accurate representations of female sexual anatomy serve to reinforce clinical information for both patients and HCPs as well as to dispel misinformation about FGM/C and the vulva. This includes the pervasive falsehood that the clitoris can be removed in its entirety during FGM/C, when in fact it is the external structures of the clitoris (the glans and sometimes part of the body of the clitoris) that may be cut through FGM/C while its internal components remain intact [23,24]. Given the complexity of clinical information imparted to patients with FGM/C and their partners, the use of visuals in consultations has been suggested to bridge linguistic and cultural barriers as well as to equip patients with understandable information about their bodies to enable informed decision-making [15,17,25-27]. Moreover, consultations often last less than 1 hour and include discussion of changes to some life-long sociocultural beliefs, with studies reporting that women with FGM/C can be overwhelmed by the amount of verbal information related to them by HCPs [17,28]. This could be improved with the use of visual aids on genital anatomy after FGM/C [15,29]. Visuals make details of available surgical procedures more accessible to patients in addition to supporting HCPs in delivering the necessary information [8,30].

**Figure 1:**
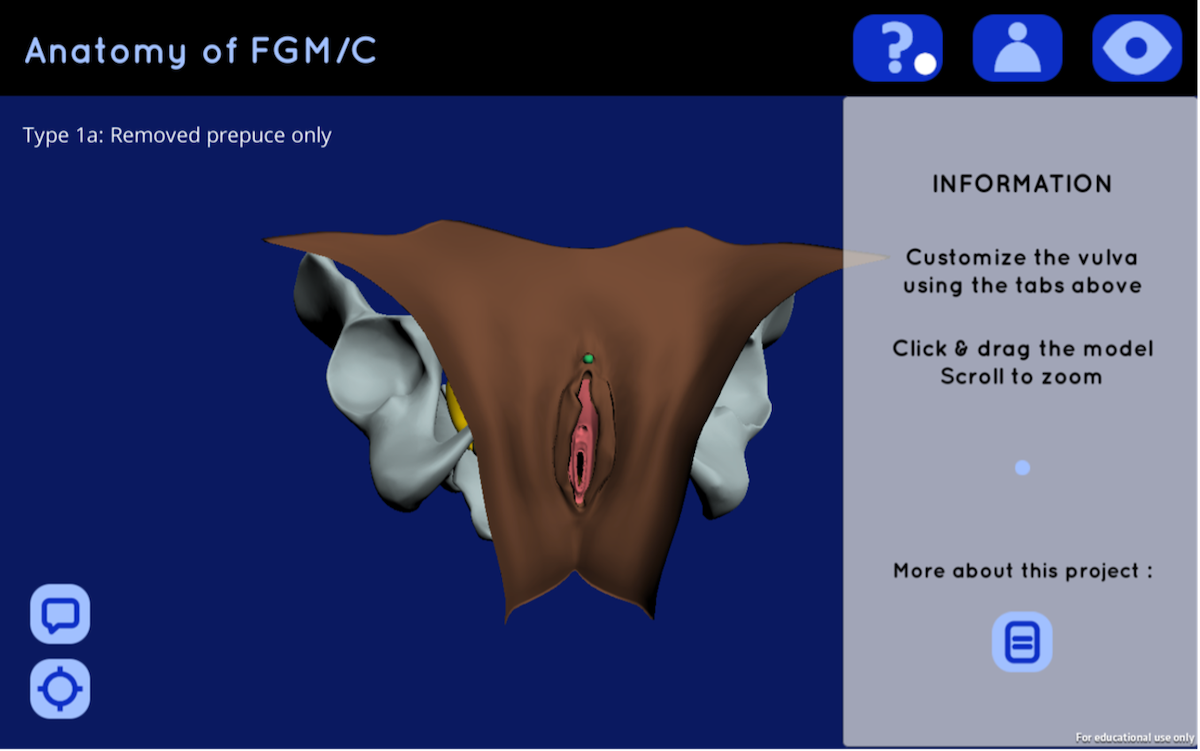
Screenshot of the 3D web app upon opening.

**Figure 2:**
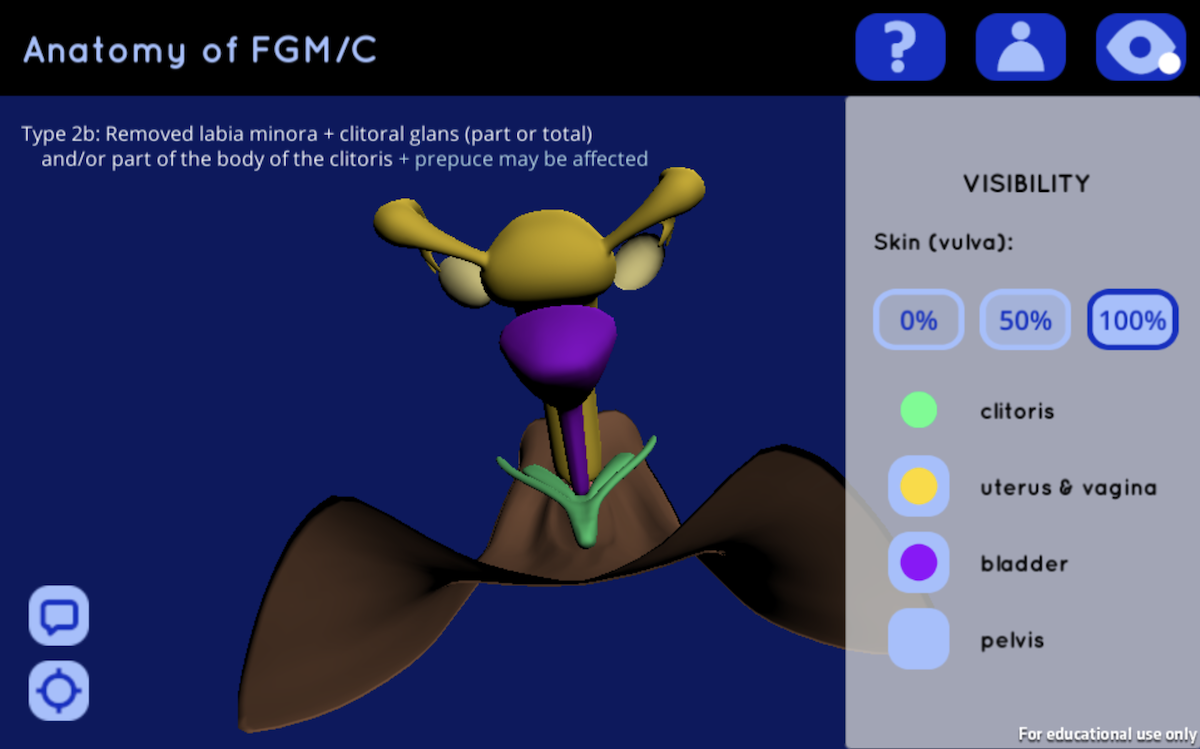
Screenshot of the 3D web app after rotation and removal of the pelvis.

Medical and patient education benefits from 3D models, which facilitate an accurate spatial understanding of anatomical structures by conveying the functional anatomy of the given area as well as the relationships between surrounding structures [31-37]. Spatial visualization is especially important to understanding the anatomy of complex areas, including the interplay of genitourinary organs in the female pelvis [38]. Moreover, interest in 3D tools is growing and educational resources about FGM/C increasingly feature 3D visualizations [30,39-41]. As such, we hypothesized that an interactive 3D visualization of female pelvic anatomy would help to describe the impact of FGM/C on physiological processes such as childbirth or penetrative intercourse with FGM/C type III more effectively than 2D visuals. Representation of the female pelvic region in 3D may also help to highlight the fact that the clitoral organ is made up of both “internal”/invisible and “external”/visible structures, of which only the latter may be affected in FGM/C (Figure 1, Figure 2). Since the inclusion of any visual aid in patient consultations requires HCP endorsement as a basis, the objective of this study was to determine whether HCPs would find an interactive 3D web app on pelvic anatomy useful in clinical discussions about FGM/C with patients.

We produced an interactive 3D visualization of FGM/C anatomy in the form of a prototypal web app for HCPs to use in patient consultations, which is available for use online and free of charge [42]. The web app features a vulvar model and its related genitourinary structures (pelvis, uterus and vagina, bladder, and clitoris), which can be manipulated to show different subtypes of FGM/C according to the typology defined by the WHO [9]. Interactive features include the options to zoom closer to or further away from as well as rotate the model, show and hide its various structures, change the skin tone of the vulva, and adjust the transparency of the skin layer to better show the anatomical structures beneath it (Figure 1, Figure 2); however, discussion of the methods used in app development is beyond the scope of this paper. HCPs experienced in care for women with FGM/C in migration-destination countries were invited to evaluate the web app in semistructured interviews conducted over a Zoom video call. Given that HCPs are the gatekeepers to health services and that the web app is primarily intended for use by HCPs, the decision was made to focus on HCP perspectives around the adoption of the 3D web app in their consultations about FGM/C as a first step to gauging their interest in such a tool.

## Methods

### Participants and Recruitment

Inclusion criteria for participants consisted of clinical experience in caring for women and girls with FGM/C in a migration-destination setting and fluency in English. Participants from different country settings and different medical specialties were recruited by author JA through purposive and convenience sampling from a pool of colleagues who run clinics for the care for women and girls with FGM/C to represent the diversity in approaches to the clinical management of FGM/C. Initial contact with HCPs was made by JA via email, with a participant information sheet and consent form attached describing the research project and assuring confidentiality of data collection and retention (see Multimedia Appendix 1). Interviews with consenting HCPs were conducted by author OMH within 1-hour time slots over Zoom video call, using a discussion guide (Multimedia Appendix 2) to ensure consistency across interviews.

### Data Collection Procedure

Semistructured interviews lasting between 25 and 55 minutes were conducted over Zoom in November 2020, which were audio- and video-recorded for data analysis. At the start of each interview, participants were asked to discuss their use of visual aids in patient consultations and their experiences with available tools. Participants were then sent a link to access the 3D web app online. They were invited to share their screens while moving through the prototype and to provide feedback by a think-aloud protocol [43] so that the primary researcher could observe and take notes on their use of the app in a “natural setting,” thus informing the analysis. Throughout this part of the evaluation, participants were encouraged to offer their feedback on the accuracy and appearance of the vulva models as well as the visual appearance, layout, and functionality of the web app. Finally, participants were asked whether and how they might use this tool with their patients, which features might be most useful to them in a consultation setting, along with any suggestions for changes or further development.

### Data Analysis

The qualitative method of analysis was chosen owing to its flexibility and potential for generating unexpected insights, which are especially valuable in lesser-researched fields and in prototypical stages of app development, with a semistructured interview protocol allowing for a more conversational approach as well as the omission and/or altering of questions if they had already been addressed in the interview. Thematic analysis of the data generated was based on the iterative 6-step method described by Braun and Clarke [44]. Interview recordings were manually transcribed by one author (OMH), which helped them to better familiarize themselves with the data generated [44]. Details on participants’ navigation through the web app based on analysis of screenshare recordings were logged next to the transcriptions of dialogue. All identifying information was removed from transcripts, with participants (N=7) randomized to a number and coded by profession in the reporting of results. Analysis of the resulting transcripts involved repeated rereading and followed an inductive approach, where themes were generated from the data through coding [44]. All data extracts were manually coded and collated within relevant code groupings, followed by sorting into higher-level topics to develop overarching themes [44]. The resulting themes were then reviewed, defined, and further refined within the research team until an agreement was reached.

### Ethical Considerations

Full ethical approval for this research was obtained through the Glasgow School of Art Postgraduate Taught Full Ethical Assessment. Participants consented to being audio- and video-recorded, after which the recordings were transcribed, anonymized, and deleted. Any information other than profession that could be used as an identifier for the participants was removed from the transcripts. Due to the small sample size, country of practice was not reported. The participant information sheet and consent form are available in Multimedia Appendix 1. Participants were not compensated in any way for taking part in this research.

## Results

### Participants and Main Themes

Out of the 16 HCPs invited to participate, 7 were recruited: one infectious disease specialist, one midwife, one pediatrician, and four obstetrician/gynecologists (OB/GYNs), all of whom were randomly assigned numerical identifiers for the analysis (Table 2). All the participants had significant experience (>10 years) in holding consultations with women and girls about FGM/C, working in multidisciplinary teams to manage treatment for patients with FGM/C in the United States (n=2), United Kingdom (n=1), Sweden (n=1), France (n=1), Belgium (n=1), and Switzerland (n=1). The authors identified three main themes from the data: (1) how visual aids are used to convey information in consultations about FGM/C, (2) how the patients with FGM/C relate to visuals describing their body, and (3) how the visual could be leveraged to improve the continuum of care for patients with FGM/C.

**Table 2:** Numerical coding of participating health care providers by profession.

Participant code	Profession
001	Infectious disease specialist
002	OB/GYN^a^
003	Midwife
004	OB/GYN
005	OB/GYN
006	Pediatrician
007	OB/GYN

^a^OB/GYN: obstetrician/gynecologist.

### What Visual Aids in Consultations About FGM/C Are Used to Convey

Participants highlighted the value of using visuals to help communicate the appearance of uncut versus cut genitalia, the functions of different vulvar structures, how the patient’s genitalia look after FGM/C, as well as the procedures of defibulation or clitoral reconstruction and how the vulva looks thereafter. However, the context and purpose of using imagery of FGM/C with patients differed among the participants. Participants leading specialist clinics for FGM/C saw patients with FGM/C frequently and had readily available visual aids for support in consultations, while others encountered fewer patients with FGM/C in their clinical practice or saw these patients in the pediatric or maternity contexts alone. Regardless, participants shared that a lot of information was often imparted to patients in their consultations about FGM/C and that visual aids of some form were necessary to describe FGM/C and its effects.

The HCPs surveyed felt that the web app and visualization would help them to address FGM/C with their patients, although several pointed out the lack of imagery for an uncut vulva. These participants felt that the addition of an uncut vulva to the visualization might be valuable for comparisons between FGM/C vulvas and those without, and would “also be useful for explaining how you do de[in]fibulation” [HCP 003], since conveying to patients what the vulva looks like underneath, after defibulation, can be “quite tricky” [HCP 003]. Otherwise, an animation or another visual way of depicting defibulation or the change between FGM/C status would be a beneficial addition to the web app. Participants appreciated that the visualization within this 3D web app emphasizes the relative size of the clitoris in the body, as well as the extent of cutting of the clitoral glans and eventual part of the body in FGM/C, “get[ting] at the fact that the entire organ is not removed” [HCP 006] and that “it’s only the top part that’s been cut” [HCP 007]. Moreover, the visualization highlights the relationship of the vulva with the internal organs (Figure 2), which is invaluable to women’s understanding of the vaginal examination and the link between the vulva, vagina, and uterus. This is particularly important in the context of pregnancy in women with infibulation (Table 1), wherein it is essential to impart that the cervix is “something that you’re trying to reach when you’re establishing whether [a patient is] in labor and why it’s preferable to defibulate antenatally” [HCP 003].

### How Patients with FGM/C Relate to Visual Aids

Participants had diverse preferences for the types of visuals they used in consultations with patients with FGM/C, whether illustrations, clinical images, or physical models, but agreed that realistic imagery that their patients can identify with is more understandable than abstract or schematic diagrams. Two OB/GYNs shared that sometimes they show clinical images of FGM/C to patients to better indicate the changes to their anatomy, with one of them presenting their patients with the “Great Wall of Vulva” [45], a compilation of diverse vulvar models that are more representative of true female genitalia than the ubiquitous and highly unrealistic vulva imagery propagated by pornography. In terms of physical models, the midwife spoke of using a series of handmade, leather-like models created by the artist Aida Silvestri [46] to help demonstrate the process of defibulation. The infectious disease specialist keeps a silicone model of a vulva [47] in their office, deeming it to be “very important and very funny” [HCP 001] to women who play with it during their consultations. One of the OB/GYNs divulged that they show a physical model of an uncut clitoris [48] to all women—whether cut or uncut—while another found the lone clitoris to be too abstract, relating that “for well-educated women it’s a little bit easier [to comprehend, but] for those who don’t know already their own body, that’s more difficult for them” [HCP 004].

As for the 3D web app, participants praised the ability to personalize the visualization to any given patient’s FGM/C type and skin tone as well as to show or hide additional organs that may be overwhelming to some women’s understanding, with three of the HCPs describing that they might display only the skin/vulva model and clitoris at first, then reveal additional organs as necessary depending on the needs of any given patient. Likewise, it was pointed out that the blue/green color used on the clitoris model in this visualization may be confusing to some patients, with a couple of participants suggesting it be adjusted to a more natural tint such as pink (Figure 1, Figure 2). All of the participants noted that one or more of the following aspects of the visualization in the web app may have negative consequences on women’s perceptions of their own genitalia in comparison: the unnaturally smooth appearance of the vulva; unrealistic symmetry in the cutting of the labia; lack of pubic hair; and, in types I and II, the exaggerated openness of the vestibule. As for the models for FGM/C type III in particular, the pediatrician cautioned that “a lot of women describe […] wanting to be ‘clean’ and […] smooth and [think that] it’s ugly if it’s all there” [HCP 006]; consequently, showing visuals that manifest these ideals may be problematic. Nevertheless, all but one of the participating HCPs were keen to adopt this 3D web app in their patient consultations, expressing that the tool “would work more easily to explain [FGM/C]” [HCP 004] than existing drawings. As with the physical models used at present, the infectious disease specialist envisioned that they would offer patients the opportunity to play with the 3D web app in consultations, prompting additional considerations for accessibility specific to patients.

Recommendations for additions to the web app included an orientation widget to better illustrate the context of the body for the patient and a higher degree of customizability to reflect natural variations in anatomy such as asymmetry as well as those related to age and parity to expand the relevance of the visualization to pediatric and obstetric contexts.

### How a 3D Web App of Genital Anatomy Could Improve the Health Care Continuum for Patients With FGM/C

Participants overwhelmingly felt that the 3D visualization is much more comprehensive and practical than the tools currently being used to communicate FGM/C, and with which HCPs “can’t do nearly as much, we’re just literally looking at a picture, and […] pointing to things” [HCP 003]. Four of the participating HCPs explicitly remarked that the web app was user-friendly, or at least that “once you’ve played with it a bit, it’s not difficult to use” [HCP 003], and could thus be easily incorporated into both consultations and health care practice in FGM/C with some small changes and additions. Suggestions for further development addressed options for user accessibility, such as further explanation on how to navigate the web app, changing the background on the visualization, or clarifying certain terminology that may be confusing to some users, such as “visibility.” Simplified descriptions of the anatomy accompanying labels for the various anatomical structures would be equally as valuable to supporting and enforcing the understanding of FGM/C in HCPs as for patients, particularly for HCPs who may have less experience with FGM/C.

Women and girls with FGM/C have different needs at different times, dictating the context in which the web app could be employed. Most importantly, the way the visualization emphasizes the fact that the tip of the clitoris is cut in FGM/C and that the rest of the clitoris remains intact “adds one piece of the puzzle that needs to be incorporated into a holistic approach that we counsel women [in sexual health] and how that informs the decision-making regarding clitoral reconstruction, for instance” [HCP 007], for both HCPs and patients with FGM/C alike. This OB/GYN spoke animatedly about how the visualization, once equipped with additional features for personalization, could be integrated into patient medical records to convey accurate information to other HCPs in the continuum of care to “help inform decision-making when the expert is no longer necessarily the primary care provider for that patient” [HCP 007].

## Discussion

### Main Findings

Overwhelmingly, the HCPs surveyed—who specialize in FGM/C and work in different migration-destination countries and with different populations, whether culturally or religiously—agreed that visuals are essential to patient care for FGM/C. Participants were adamant about using visual aids in consultations with their patients, impressed with the customizability offered by this 3D web app, and keen to adopt it as part of their health care practice. Participants drew from clinical experiences in their discussions of how this tool could improve not only patient consultations but other facets across the continuum of health care for women with FGM/C. Their observations around successful visuals, including what works in this tool and what needs improvement, offer insight into where the focus should be in developing more effective tools to support patient care in FGM/C: that the visual conveys what it needs to, how the patient relates to and thus comprehends the visual, and how the visual could be integrated into the wider continuum of care for patients with FGM/C. All but one of the participants expressed interest in using this prototypical visualization to supplement their patient consultations, acknowledging the value of using an interactive visual tool in FGM/C care.

### Strengths and Limitations

This study is the first of its kind to develop a prototype for an interactive 3D visualization of the anatomy after FGM/C and have it evaluated by HCPs specializing in the field for feedback on its value and practicality for use in patient consultations. Although user interviews should ideally be conducted prior to design to determine the users’ needs and how to address them [49], limited resources led this study to develop the 3D web app based on research gaps identified in a review of the literature, including the fact that no such web app existed for HCP use in patient consultations about FGM/C at the time of writing. Furthermore, the use of visual aids had already been suggested by researchers to mitigate barriers in communication between HCPs and patients with FGM/C [26,27].

The qualitative nature of this study allowed for comprehensive and contextual feedback, with the tradeoff being the small pool sample of potential users. Nevertheless, the semistructured interview protocol allowed participants the opportunity to share their experiences using various visual tools, elaborate on their strengths and weaknesses, and then measure them up against the web app prototype developed for the study. Reasons for not participating in the study were not identified, but may include time burden, apprehension around the use of external visuals of FGM/C with patients, or aversion to using technology in clinical practice [50-52]. The latter two may underpin one participant’s skepticism around implementing the web app in their clinical practice. Echoing similar concerns from other participants about enforcing idealized body image and beauty standards on women and girls with FGM/C, this OB/GYN was apprehensive about the potential psychosexual effects of showing patients imagery of vulvas that are not their own, opting instead for using a mirror or manually sketching a given patient’s vulva if they were reluctant to look at their reflection. These findings hint at the wider discourse around body image and beauty standards in FGM/C and beyond [53].

While this research does not yet include feedback from patients, which is recognized as an essential component to the further development of any such tool, HCPs are the intended primary users of this web app. As such, their endorsement is vital to determining whether it could be implemented in their consultation practice and their initial feedback is an important first step. The HCPs surveyed in this study had different medical specialties and were based in different high-income migration-destination countries, offering varied perspectives on the tool’s value to their clinical practice. Although participants could only be coded by profession in the data analysis to protect their identities per the consent obtained for this study, stratifying HCPs by country of practice, especially in a larger study population, may give further insight to the data in line with differing laws and conventions related to the reporting and care for FGM/C around the world. Despite differences in preference for any specific type of visual aid, all the participating HCPs agreed that imagery of any sort is essential to communicating health information to patients about FGM/C.

Furthermore, the accessibility and customizability offered by digital models are invaluable to their ease of use in health care practices and portrayal of anatomy that is representative of any given patient, which is limited with 3D printed models. As the web app is hosted online, it can be accessed from anywhere in the world using a computer. This functionality is especially useful for HCPs who may not see patients with FGM/C often enough to keep physical models of vulvas affected by FGM/C in their office. The digital nature of the tool also allows for tailoring to specific settings and contexts beyond patient consultations, such as for personal use by patients or in HCP education. In fact, over 2 years after the interviews, the participant who had expressed reluctance about using the web app with patients contacted author JA with additional feedback: they had taken part in a trial related to FGM/C and felt that the web app would be useful in medicolegal settings, such as to describe the clitoris and FGM/C to judges and lawyers.

### Interpretation

All patients are entitled to relevant health information, especially when providing consent for medical and surgical treatment, as well as multidisciplinary care for any gynecological, psychological, and psychosexual issues, whether related to FGM/C or not. Positive health care experiences and clinical outcomes are linked to shared decision-making between patients and HCPs, which requires a mutual understanding of clinical and health information that can be effectively relayed using visuals [4,17]. For patients, increased bodily awareness encourages better self-care, sexual experience, and understanding of the harms of FGM/C, affording women the autonomy that translates into improved health and well-being. For HCPs, visual tools help support the communication of complex clinical information to patients and may also serve to reinforce their own understanding of sexual and genitourinary anatomy.

The variability across genitalia—irrespective of FGM/C type—cannot be accurately represented by any one single visual. Fortunately, features for highlighting vulvar individuality can be implemented into the web app, such as including a gallery of imagery featuring a variety of vulvas—such as those from the recently published illustrative guide on FGM/C [54]—or an option for manipulating the 3D model itself. The addition of a function for adjusting the 3D model in real time [55] may require further consideration of HCPs’ technical abilities and willingness to use it as such [56], but can be circumvented with the inclusion of presets addressing the more common natural variances in genital structures. Although offering a higher degree of authenticity in the visuals, further options for its personalization, as well as use of this tool to supplement already comprehensive, culturally sensitive communications from the HCP, should mitigate concerns around the effects of showing patients standardized imagery of different FGM/C types versus their own vulvas; however, this topic deserves further exploration.

### Conclusions

This study gives insight into the use of a 3D web app visualizing genital anatomy to support the communication of clinical information in patient consultations about FGM/C. The findings support the need for a wider conversation following more in-depth research about the best ways of using visuals to supplement the provision of health care to women and girls with FGM/C. Upon evaluation of the 3D web app prototype, HCPs with expertise in the field compellingly deemed it a valuable tool for conferring information about FGM/C in addition to providing comprehensive and multidisciplinary care to women living with FGM/C. Given the positive feedback from HCPs in this study, the next step would be to have the web app evaluated in a consultation setting together with HCPs and patients to test the usability and effectiveness of the app in conveying clinical information about FGM/C. As FGM/C is performed at a young age, the tool should also be adapted for girls. A younger population may even be more receptive to a digital medium for communicating health information than older patients, although tailoring any web app to reduce barriers to use by older patients is equally important [57,58].

Beyond use in patient consultations, the 3D visuals of genitalia with FGM/C created for this web app can be readily incorporated into other aspects of health care and information dissemination, including FGM/C prevention by debunking myths about the clitoris, the vulva, the vagina, the hymen, and sexuality overall, or by helping to document FGM/C status in medical records to support the continuum of care for and mandatory reporting of FGM/C, as well as in medicolegal and policy settings [26,30,59]. Further options for customizability, accessibility, and integration of this tool into the continuum of care for women and girls with FGM/C should be explored.

## Appendix

Multimedia Appendix 1 Participant information sheet and consent form.

Multimedia Appendix 2 Discussion guide.

## Abbreviations

FGM/C: female genital mutilation or cutting
HCP: health care provider
OB-GYN: obstetrician-gynecologist
WHO: World Health Organization

## Data Availability

The data sets generated and analyzed during this study are available from the corresponding author on reasonable request.

## References

[1] Dawson AJ, Turkmani S, Varol N, Nanayakkara S, Sullivan E, Homer CSE (2015). Midwives' experiences of caring for women with female genital mutilation: insights and ways forward for practice in Australia. Women Birth.

[2] Abdulcadir J, Rodriguez MI, Say L (2015). Research gaps in the care of women with female genital mutilation: an analysis. BJOG.

[3] Evans C, Tweheyo R, McGarry J, Eldridge J, McCormick C, Nkoyo V, Higginbottom GMA (2017). What are the experiences of seeking, receiving and providing FGM-related healthcare? Perspectives of health professionals and women/girls who have undergone FGM: protocol for a systematic review of qualitative evidence. BMJ Open.

[4] Evans C, Tweheyo R, McGarry J, Eldridge J, Albert J, Nkoyo V, Higginbottom G (2019). Improving care for women and girls who have undergone female genital mutilation/cutting: qualitative systematic reviews. Health Serv Deliv Res.

[5] Johnson-Agbakwu CE, Manin E (2021). Sculptors of African women's bodies: forces reshaping the embodiment of female genital cutting in the west. Arch Sex Behav.

[6] Lurie JM, Weidman A, Huynh S, Delgado D, Easthausen I, Kaur G (2020). Painful gynecologic and obstetric complications of female genital mutilation/cutting: a systematic review and meta-analysis. PLoS Med.

[7] Abdulcadir J, McLaren S, Boulvain M, Irion O (2016). Health education and clinical care of immigrant women with female genital mutilation/cutting who request postpartum reinfibulation. Int J Gynaecol Obstet.

[8] Smith H, Stein K (2017). Health information interventions for female genital mutilation. Int J Gynaecol Obstet.

[9] (2018). Care of girls and women living with female genital mutilation: a clinical handbook. World Health Organization.

[10] Evans C, Tweheyo R, McGarry J, Eldridge J, Albert J, Nkoyo V, Higginbottom G (2019). Crossing cultural divides: a qualitative systematic review of factors influencing the provision of healthcare related to female genital mutilation from the perspective of health professionals. PLoS One.

[11] Abdulcadir J, Say L, Pallitto C (2017). What do we know about assessing healthcare students and professionals' knowledge, attitude and practice regarding female genital mutilation? A systematic review. Reprod Health.

[12] Marea CX, Warren N, Glass N, Ahmed W, Pallitto CC (2023). Advancing the measurement of knowledge, attitudes and practices of health workers who care for women and girls who have undergone female genital mutilation/ cutting (FGM/C): A qualitative exploration of expert opinion. PLoS One.

[13] Connelly E, Murray N, Baillot H, Howard N (2018). Missing from the debate? A qualitative study exploring the role of communities within interventions to address female genital mutilation in Europe. BMJ Open.

[14] McCauley M, van den Broek N (2019). Challenges in the eradication of female genital mutilation/cutting. Int Health.

[15] Brady SS, Connor JJ, Chaisson N, Sharif Mohamed F, Robinson BBE (2021). Female genital cutting and deinfibulation: applying the theory of planned behavior to research and practice. Arch Sex Behav.

[16] (2016). WHO guidelines on the management of health complications from female genital mutilation. World Health Organization.

[17] Young J (2019). Female genital cutting in immigrant children—considerations in treatment and prevention in the United States. Curr Sex Health Rep.

[18] Abdulcadir J, Manin E, Earp BD, Ferguson EMN, O'Dey DM, Johnson-Agbakwu CE (2022). Rethinking reconstruction: ethical standards and practice guidelines as a prerequisite to clitoral reconstruction following female genital mutilation/cutting. Aesthet Surg J.

[19] Klein E, Helzner E, Shayowitz M, Kohlhoff S, Smith-Norowitz TA (2018). Female genital mutilation: health consequences and complications-a short literature review. Obstet Gynecol Int.

[20] Easwaran L, Eidelson SA, Jain A, Akaniru O, Rattan R, Thaller S (2022). Female genital mutilation: treatment updates and the need for education. J Craniofac Surg.

[21] Sharif Mohamed F, Wild V, Earp BD, Johnson-Agbakwu C, Abdulcadir J (2020). Clitoral reconstruction after female genital mutilation/cutting: a review of surgical techniques and ethical debate. J Sex Med.

[22] (2018). Female genital mutilation: Standards for training healthcare professionals. NHS England.

[23] Abdulcadir J, Botsikas D, Bolmont M, Bilancioni A, Djema DA, Bianchi Demicheli F, Yaron M, Petignat P (2016). Sexual anatomy and function in women with and without genital mutilation: a cross-sectional study. J Sex Med.

[24] Johnsdotter S (2018). The impact of migration on attitudes to female genital cutting and experiences of sexual dysfunction among migrant women with FGC. Curr Sex Health Rep.

[25] Johansen REB (2017). Undoing female genital cutting: perceptions and experiences of infibulation, defibulation and virginity among Somali and Sudanese migrants in Norway. Cult Health Sex.

[26] Shaikh H, McDonnell KA (2020). Review of web-based toolkits for health care practitioners working with women and girls affected by or at risk of female genital mutilation/cutting. J Prim Care Community Health.

[27] Bansal S, Breckwoldt M, O'Brien Green S, Mbugua S (2013). Female genital mutilation. Information for health-care professionals working in Ireland. 2nd edition.

[28] Turkmani S, Homer CSE, Dawson A (2019). Maternity care experiences and health needs of migrant women from female genital mutilation-practicing countries in high-income contexts: A systematic review and meta-synthesis. Birth.

[29] Jacoby SD, Lucarelli M, Musse F, Krishnamurthy A, Salyers V (2015). A mixed-methods study of immigrant Somali women's health literacy and perinatal experiences in Maine. J Midwifery Womens Health.

[30] BAADON: take your power back.

[31] Fredieu JR, Kerbo J, Herron M, Klatte R, Cooke M (2015). Anatomical models: a digital revolution. Med Sci Educ.

[32] Canbazoglu E, Salman YB, Yildirim ME, Merdenyan B, Ince IF (2016). Developing a mobile application to better inform patients and enable effective consultation in implant dentistry. Comput Struct Biotechnol J.

[33] Jones DB, Sung R, Weinberg C, Korelitz T, Andrews R (2016). Three-dimensional modeling may improve surgical education and clinical practice. Surg Innov.

[34] Keenan I, Ben Awadh A (2019). Integrating 3D visualisation technologies in undergraduate anatomy education. Adv Exp Med Biol.

[35] Knight O, Carter CR, Loranger B, Rea PM (2019). Recommended workflow methodology in the creation of an interactive application for patient's diagnosed with pancreatic cancer. Adv Exp Med Biol.

[36] Triepels CPR, Smeets CFA, Notten KJB, Kruitwagen RFPM, Futterer JJ, Vergeldt TFM, Van Kuijk SMJ (2020). Does three-dimensional anatomy improve student understanding?. Clin Anat.

[37] Azer SA, Azer S (2016). 3D anatomy models and impact on learning: a review of the quality of the literature. Health Profess Educ.

[38] Kiesel M, Beyers I, Kalisz A, Joukhadar R, Wöckel A, Herbert S, Curtaz C, Wulff C (2022). A 3D printed model of the female pelvis for practical education of gynecological pelvic examination. 3D Print Med.

[39] Ministère chargé de l'égalité (2023). L'anatomie génitale. YouTube.

[40] Ministère chargé de l'égalité (2023). Les trois types d'excisions. YouTube.

[41] Ministère chargé de l'égalité (2023). La chirurgie réparatrice de l'excision. YouTube.

[42] Anatomy of FGM/C webapp. oliviamayvisuals.

[43] Wood LE (1997). Semi-structured interviewing for user-centered design. Interactions.

[44] Braun V, Clarke V (2006). Using thematic analysis in psychology. Qual Res Psychol.

[45] The Great Wall of Vulva.

[46] (2016). Unsterile clinic: silhouettes of FGM survivors. Hackney Citizen.

[47] Shop. SEX-ED+.

[48] Fillod O Clitoris imprimés en 3D. PIY3D.

[49] Thornton P (2019). How to conduct user interviews. UX Collective.

[50] Negrin KA, Slaughter SE, Dahlke S, Olson J (2022). Successful recruitment to qualitative research: a critical reflection. Int J Qual Methods.

[51] Yousef CC, Salgado TM, Burnett K, Aldossary I, McClelland LE, Alhamdan HS, Khoshhal S, Aldossary I, Alyas OA, DeShazo JP (2023). Perceived barriers and enablers of a personal health record from the healthcare provider perspective. Health Informatics J.

[52] Jarva E, Oikarinen A, Andersson J, Tuomikoski A, Kääriäinen M, Meriläinen M, Mikkonen K (2022). Healthcare professionals' perceptions of digital health competence: a qualitative descriptive study. Nurs Open.

[53] Boddy J (2020). Re-thinking the zero tolerance approach to FGM/C: the debate around female genital cosmetic surgery. Curr Sex Health Rep.

[54] Abdulcadir J, Guedj NS, Yaron M (2022). Female genital mutilation/cutting in children and adolescents: illustrated guide to diagnose, assess, inform and report.

[55] The Sims 4. Electronic Arts.

[56] Yoon D, Kim K (2013). Interactive evolution of 3D models based on direct manipulation for video games. Procedia Comput Sci.

[57] Byambasuren O, Beller E, Hoffmann T, Glasziou P (2020). Barriers to and facilitators of the prescription of mHealth apps in Australian general practice: qualitative study. JMIR Mhealth Uhealth.

[58] Wildenbos GA, Peute L, Jaspers M (2018). Aging barriers influencing mobile health usability for older adults: a literature based framework (MOLD-US). Int J Med Inform.

[59] Abdulcadir J, Brockmann C, Dewaele R, Holuszko O WebApp: Let's talk FGM/C. Sciences, Sexes, Identités. Université de Genève.

