# Supplementary material for: Health Care Providers’ Readiness to Adopt an Interactive 3D Web App in Consultations About Female Genital Mutilation/Cutting: Qualitative Evaluation of a Prototype
Source: JMIR Form Res. 2023 Sep 28;7:e44696. doi: 10.2196/44696 (PMC10570893; doi:10.2196/44696)
Supplement: Multimedia Appendix 1 [file formative_v7i1e44696_app1.pdf]

SCHOOL OF SIMULATION AND VISUALISATION
THE GLASGOW SCHOOL OF ART

University of Glasgow

Participant Information Sheet

PROJECT: Development of an interactive, digital 3D model of female pelvic anatomy specific to FGM/C to facilitate woman-centred care.

You have been invited to help with this study based on your role as a health care provider with experience in caring for women living with FGM/C. Your involvement could make a significant contribution to the research. Please read this information sheet before deciding whether or not you would like to participate.
If you do not wish to participate, you do not have respond to this request.
If you would like to participate, please

1. **read this information sheet;**
2. **sign the consent form and send it to 2513271h@student.gla.ac.uk;**
3. **select a timeslot for a 30-60 min evaluation session here: https://doodle.com/poll/r7grvrkhznwxscid.**

You will receive an email reminder with the Zoom meeting link one day before your scheduled session. If you have any questions about the study or if none of the proposed timeslots work for you, please contact Olivia.

CONTEXT & PURPOSE

This study was developed as a response to the need for better communication between health care practitioners (HCPs) and women affected by FGM/C as well as involvement of women in their treatment plans.[1–4] Many women living with FGM/C have reported feeling disengaged from HCP decision-making, underinformed about available treatment options, as well as overwhelmed by the amount of FGM/C-related information provided in consultations.[2–5] The use of visual aids in consultations about FGM/C may help to transcend language barriers and empower women with understandable knowledge about their anatomy.[6–9]

The purpose of the study is to explore whether an interactive application on the anatomy of FGM/C in 3D may be useful to health care practitioners in consultations with women affected by FGM/C. Your feedback may contribute to further development and possible implementation of this or similar tools in health care practice and/or in education about FGM/C.

WHAT DO I NEED TO DO?

You will be asked to evaluate a prototype for a simple app with a 3D visualisation of female pelvic anatomy and vulvas affected by FGM/C. The session will take place over recorded Zoom call. You may stop at any point.

If you agree to participate, you will need:

- a quiet location of your choice (ie. workplace or home office);
- a desktop or laptop computer with mouse, stable internet connection and Zoom installed – please ensure that your microphone (and optional camera) is fully functioning in Zoom by testing here: https://zoom.us/test.

WILL MY TAKING PART IN THE STUDY BE KEPT CONFIDENTIAL?

All information provided by you will be kept confidential and within the research team. Recordings will be safely stored and accessible by the research team alone, for analyses and transcription, after which the files will be deleted. Any personal information that could identify you will be removed or changed before results are made public. All responses to our questions will be anonymized, i.e. no personal details relating to you will be recorded anywhere except your profession (eg. OB/GYN, surgeon, midwife) which will be used as an identifier. Information emanating from the evaluation will only be made public in a completely unattributable format or at the aggregate level (may include direct quotes) in order to ensure that no participant will be identified. Under no circumstances will identifiable responses be provided to any other third party. The anonymized data collected will be retained for 5 years for future research or potential further development of the project. You may withdraw from the study at any time -- data collected about you will be deleted unless already anonymized/aggregated, in which case it will not be possible to identify and remove your contribution. If you disclose information that may result in you or anyone else being put at risk of harm, we may have to inform the appropriate authorities.

If you have any complaints about this study**,** please contact any member of the research team or the Head of Postgraduate Programmes, Daniel Livingstone: d.livingstone@gsa.ac.uk.

RESEARCH TEAM

Olivia Holuszko (Lead Researcher)	2513271h@student.gla.ac.uk
Daisy Abbott (Glasgow School of Art)	d.abbott@gsa.ac.uk
Dr. Jasmine Abdulcadir (Geneva University Hospitals)	jasmine.abdulcadir@hcuge.ch
Dr. Jenny Clancy (University of Glasgow)	jenny.clancy@glasgow.ac.uk

REFERENCES

1. Dawson AJ, Turkmani S, Varol N, Nanayakkara S, Sullivan E, Homer CSE. Midwives' experiences of caring for women with female genital mutilation: Insights and ways forward for practice in Australia. *Women and Birth*. 2015;28(3):207-214. doi:10.1016/j.wombi.2015.01.007
2. Smith H, Stein K. Health information interventions for female genital mutilation. *Int J Gynaecol Obstet*. 2017;136:79-82. doi:10.1002/ijgo.12052
3. Evans C, Tweheyo R, McGarry J, et al. *Crossing Cultural Divides: A Qualitative Systematic Review of Factors Influencing the Provision of Healthcare Related to Female Genital Mutilation from the Perspective of Health Professionals*. Vol 14.; 2019. doi:10.1371/journal.pone.0211829
4. Turkmani S, Homer CSE, Dawson A. Maternity care experiences and health needs of migrant women from female genital mutilation–practicing countries in high-income contexts: A systematic review and meta-synthesis. *Birth*. 2019;46(1):3-14. doi:10.1111/birt.12367
5. Evans C, Tweheyo R, McGarry J, et al. What are the experiences of seeking, receiving and providing FGM-related healthcare? Perspectives of health professionals and women/girls who have undergone FGM: Protocol for a systematic review of qualitative evidence. *BMJ Open*. 2017;7(12). doi:10.1136/bmjopen-2017-018170
6. Brady SS, Connor JJ, Chaisson N, Sharif Mohamed F, Robinson B "Bean" E. Female Genital Cutting and Deinfibulation: Applying the Theory of Planned Behavior to Research and Practice. *Arch Sex Behav*. 2019. doi:10.1007/s10508-019-1427-4
7. Young J. Female Genital Cutting in Immigrant Children—Considerations in Treatment and Prevention in the United States. *Curr Sex Heal Reports*. 2019;11(2):108-114. doi:10.1007/s11930-019-00200-3
8. Johansen REB. Undoing female genital cutting: perceptions and experiences of infibulation, defibulation and virginity among Somali and Sudanese migrants in Norway. *Cult Heal Sex*. 2017;19(4):528-542. doi:10.1080/13691058.2016.1239838
9. Shaikh H, McDonnell KA. Review of Web-Based Toolkits for Health Care Practitioners Working With Women and Girls Affected by or at Risk of Female Genital Mutilation / Cutting. 2020. doi:10.1177/2150132720935296

Research Consent Form

Please initial the boxes on the right to agree to the following: ***Initials***

1. I confirm that I have read and understand the participant information sheet for this study;
2. I have had an opportunity to consider the information, ask questions, and have had these questions answered satisfactorily;
3. I agree to having my audio recorded (video optional) and understand that these recordings will be deleted after transcription;
4. I understand that any information disclosed and/or published will remain anonymous, and that transcripts may be edited to remove identifying information;
5. I agree to anonymized transcriptions being made publicly available in publications, presentations, reports, or examinable format (dissertation or thesis);
6. I agree to the results of this study being published as part of a Master's thesis, as well as used for future publication, research, or teaching purposes;
7. I agree to receiving essential communication about the study, such as Zoom link to evaluation session, by email (*see below**);
8. I agree to take part in this study.

Name of participant	Date	Signature

Researcher	Date	Signature

** Please return this consent form to 2513271h@student.gla.ac.uk using the email address that you agree to being contacted at for the duration of the study.*

☐ I would like to receive an email follow-up regarding the results of the study.
