# Supplementary material for: Health Care Providers’ Readiness to Adopt an Interactive 3D Web App in Consultations About Female Genital Mutilation/Cutting: Qualitative Evaluation of a Prototype
Source: JMIR Form Res. 2023 Sep 28;7:e44696. doi: 10.2196/44696 (PMC10570893; doi:10.2196/44696)
Supplement: Multimedia Appendix 2 [file formative_v7i1e44696_app2.docx]

## Appendix 2: Discussion Guide

**Preparation:**

- Enable screensharing for participants in Zoom
- Set phone and laptop to “Do not disturb”
- Technical checks

**Introduction:**

- Thank you for your time and participation
- Introductions
- Research context and purpose
- Agenda for the session:
  - Contextual questions prior to webapp evaluation
  - Evaluation of the webapp through screenshare
- Reminder about time allotted for the session
- Reminder of option to pause or withdraw at any point
- Reminder that researcher will stay on the line for the full hour
- Consent check and prompt for questions
- Confirm consent for video- and audio-recording
- Start video- and audio-recording

**Contextual questions:**

- What is your profession and what does a typical work day look like for you?
  - In the context of FGM/C, what is your role in caring for patients?
  - When you have consultations with FGM/C patients, what is usually the topic of these conversations?
  - Would you mind briefly walking me through what a typical consultation with a woman affected by FGM/C might look like?
  - What kind of information are patients looking for and how you provide it?
- Do you discuss anatomy with FGM/C patients and do you use any visual aids like illustrations or physical models?
  - Would you mind telling me about more about them? What kind – physical or digital? Were/are they FGM/C-specific?
  - Why and how (what about them) were/are they useful and why/why not?

**Transition from questions to evaluation:**

- Reminder that webapp is a prototype so some features will work while others may not, and honest feedback is especially appreciated
- Reminder to participant to use think-aloud protocol, i.e. talking while using the webapp, whether just thoughts or specific comments
- Reminder that it is the webapp that is being tested, not the participant, so there is no right or wrong
- Request screenshare
  - Click the green button in the center/middle at the bottom of our video chat, and then Zoom will ask you which screen to share.
- Share link and password to webapp in Zoom chat
  - Request to open webapp in fullscreen using button in the bottom-right corner below the app window

**Webapp evaluation:**

- Offer participant 2-3 minutes to look around
  - Remind participant to think-aloud
- Now that you’ve had a look, what are your overall impressions of the webapp?
  - Could you talk about some of the things that you like?
  - What about the things that you don’t like as much?
- What do you think of the app? The layout, the organization - how it looks and works?
- What do you think of the language used, the wording?
- What do you think of the vulvas and how they look?
- Do you think that this tool could be useful to you in consultations with FGM/C patients? How so or why not?
- Could I ask you to imagine that you’re using this tool with a patient. How would you use it, and which of the features do you think you would use?
  - I notice you didn’t use [feature], is that something you would use with a patient?
  - How do you think this tool might help patients to better understand their anatomy and FGM/C?
- How could this tool be changed or improved to make it more useful to your work?
- Any final comments?
  - Reminder to email any additional thoughts or comments if they arise

**Close:**

- Reminder that the participant can withdraw at any point until the data is aggregated, at which point specific contributions cannot be identified and removed
- Any questions?
  - Reminder to contact the research team regarding any questions or concerns about the study
- Thank you for your time and participation
